# Mortality Rates of Minor vs Major Lower Extremity Amputations in Diabetic Patients

**DOI:** 10.15388/Amed.2025.32.2.3

**Published:** 2025-12-30

**Authors:** Karolis Strašunskas, Vėtra Markevičiūtė

**Affiliations:** 1 Lithuanian University of Health Sciences, Faculty of Medicine, Kaunas, Lithuania; 2 Department of Orthopaedic Surgery, Kaunas Clinics, Kaunas, Lithuania

**Keywords:** diabetes mellitus, lower extremity amputations, mortality rates, cukrinis diabetas, apatinių galūnių amputacijos, mirtingumo rodikliai

## Abstract

**Background:**

Mortality rates between minor (e.g., removal of toes or part of the foot) and major (e.g., below- or above-the-knee) lower extremity amputations (LEA) vary significantly in diabetic patients. Other factors, including survival rates, rehabilitation outcomes, and healthcare burden, are also notably impacted by the type of amputation performed.

**Objective:**

This narrative review aims to analyze and compare mortality rates following minor and major LEA in diabetic patients, by highlighting key risk factors and their impact on patient outcomes.

**Methods:**

A narrative review of existing literature was conducted by using searches of *PubMed* and *Google Scholar*. Studies reporting mortality rates, risk factors, comorbidities, functional outcomes, and management strategies among diabetic patients undergoing minor and major LEA were included.

**Results:**

Major LEA is associated with significantly higher short- and long-term mortality rates, with five-year survival ranging from 10% to 48%, compared to 29% to 69% for minor LEA. The key risk factors for mortality include chronic renal disease, peripheral arterial disease, sepsis, and poor glycemic control. While minor LEA offers better survival rates, it carries a higher risk of progression to major amputation if diabetes-related complications persist.

**Conclusion:**

The findings highlight the critical importance of early intervention, strict glycemic control, and multidisciplinary care to improve the survival and quality of life in diabetic patients undergoing LEA. Limb preservation strategies should be prioritised whenever possible, as minor amputations lead to better long-term outcomes. Future research should focus on refining risk stratification and optimizing rehabilitation programs to enhance patient prognosis post-amputation.

## Abstract (it)

**Įvadas:**

Diabetu sergančių pacientų mirtingumo dėl nedidelių (pvz., pirštų ar pėdos dalies pašalinimas) ir didelių (pvz., apatinių galūnių amputacijos (angl. *lower extremity amputations* (LEA)) žemiau ar aukščiau kelio) amputacijų rodikliai labai skiriasi. Kitiems veiksniams, įskaitant išgyvenamumą, reabilitacijos rezultatus ir sveikatos priežiūros naštą, taip pat daro didelę įtaką atliktos amputacijos tipas.

**Tikslas:**

Šios apžvalgos tikslas – išanalizuoti ir palyginti cukriniu diabetu sergančių pacientų mirtingumo po nedidelių ir didelių LEA rodiklius, išryškinti pagrindinius rizikos veiksnius ir jų poveikį pacientų gydymo rezultatams.

**Metodai:**

Naudojant *PubMed* ir *Google Scholar* paieškos sistemas atlikta esamos literatūros apžvalga. Buvo įtraukti tyrimai, kuriuose aprašytas cukriniu diabetu sergančių pacientų, kuriems atlikta mažoji ir didžioji LEA, mirtingumas, rizikos veiksniai, gretutinės ligos, funkciniai rezultatai ir valdymo strategijos.

**Rezultatai:**

Didelė LEA yra susijusi su gerokai didesniu trumpalaikiu ir ilgalaikiu mirtingumu: pacientų penkerių metų išgyvenamumas svyruoja nuo 10 % iki 48 %, o mažos LEA atveju – nuo 29 % iki 69 %. Pagrindiniai mirtingumo rizikos veiksniai yra lėtinė inkstų liga, periferinių arterijų liga, sepsis ir prasta glikemijos kontrolė. Nors nedidelės LEA išgyvenamumo rodikliai yra geresni, tačiau, jei išlieka su diabetu susijusios komplikacijos, yra didesnė rizika, kad ji gali peraugti į didelę amputaciją.

**Išvados:**

siekiant pagerinti cukriniu diabetu sergančių pacientų, kuriems atliekama LEA, išgyvenamumą ir gyvenimo kokybę, labai svarbi ankstyva intervencija, griežta glikemijos kontrolė ir daugiadalykė priežiūra. Kai tik įmanoma, pirmenybė turėtų būti teikiama galūnių išsaugojimo strategijoms, nes nedidelės amputacijos lemia geresnius ilgalaikius rezultatus. Ateityje atliekant mokslinius tyrimus daugiausia dėmesio turėtų būti skiriama rizikos stratifikacijai tobulinti ir reabilitacijos programoms optimizuoti, kad pagerėtų pacientų prognozė po amputacijos.

## Introduction

Diabetes mellitus is one of the most prevalent chronic diseases worldwide, often leading to complications such as polyneuropathy and *Peripheral Vascular Disease* (PVD). These complications substantially increase the risk of developing foot ulcerations, which, if not properly managed, can result in *Lower Extremity Amputations* (LEA). LEA represents a serious consequence, with both minor (e.g., removal of toes or part of the foot) and major (e.g., below- or above-the-knee) amputations posing distinct challenges for patient survival, rehabilitation, and the quality of life. These challenges impose a significant burden on both patients and healthcare systems globally. Various clinical, social, and economic factors influence the decision-making process surrounding amputation, making it crucial to understand the associated mortality rates. This understanding is essential for improving the patient care, optimizing treatment strategies, and enhancing long-term outcomes.

The pathophysiology of diabetes plays a significant role in the progression to LEA. In *Type 2 Diabetes Mellitus* (T2DM), defective insulin secretion by pancreatic β-cells and *Insulin Resistance* (IR) in insulin-sensitive tissues such as muscle, liver, and adipose tissue lead to chronic hyperglycemia. This condition is exacerbated by chronic inflammation, oxidative stress, and mitochondrial dysfunction, which impair glucose regulation and β-cell function [1]. In *Type 1 Diabetes Mellitus* (T1DM), autoimmune destruction of β-cells, marked by islet-cell autoantibodies, leads to progressive dysfunction, with symptomatic hyperglycemia emerging once 80–90% of the β-cell function is lost. Persistent autoimmunity overwhelms β-cell regeneration, as these cells have limited regenerative capacity. Autoantibodies serve as biomarkers but do not directly cause T1DM [2]. Both T1DM and T2DM contribute to microvascular complications such as retinopathy, nephropathy, and neuropathy, as well as macrovascular complications like PVD, all of which increase the likelihood of foot ulcers and subsequent amputations [1], [2]. Additionally, mitochondrial dysfunction exacerbates complications by disrupting energy metabolism, increasing lipid accumulation, reducing glucose uptake, and amplifying oxidative stress. Gut dysbiosis, characterized by an imbalance in gut microbiota, further impairs inflammation and insulin sensitivity, increasing the risk of diabetic complications [1].

This literature review aims to analyze and compare mortality rates following minor and major LEA in diabetic patients. By integrating existing literature, epidemiological data, and clinical insights, this study seeks to identify the factors influencing mortality outcomes and to explore opportunities to enhance the patient care. Furthermore, disparities in mortality rates across demographic groups, including the age, gender, socioeconomic status, and comorbidities, will be examined to better understand the complexities of managing diabetic complications in diverse populations.

The significance of this research lies in its potential to inform healthcare providers, policymakers, and researchers about the mortality risks associated with minor and major LEA in diabetic patients. By identifying high-risk groups and modifiable risk factors, clinicians can better tailor interventions to mitigate these risks, improve the survival rates, and enhance the quality of life for patients. Understanding the impact of the amputation type on mortality outcomes can guide treatment decisions, rehabilitation strategies, and multidisciplinary care approaches, ultimately addressing the challenges posed by diabetic complications and improving long-term patient outcomes.

## Materials and Methods

### Search Design

This review was conducted as a structured narrative literature review with the primary aim of analyzing and comparing the mortality outcomes following minor and major lower extremity amputations (LEA) in diabetic patients. A comprehensive but non-systemic literature search was conducted, selecting studies that evaluated mortality rates, reamputation risk, postoperative complications, energy expenditure, and long-term functional outcomes following LEA in diabetic populations.

### Databases and Search Terms

- PubMed
- Google Scholar

The searches were performed by using the following key terms individually and in combination with Boolean operators (AND, OR):

- “Mortality rates minor lower extremity amputation”;
- “Mortality rates major lower extremity amputation”;
- “Diabetes mellitus lower extremity amputation”;
- “Reamputation risk diabetic patients”;
- “Functional outcomes lower extremity amputation”;
- “Energy expenditure lower limb amputation”;
- “Prosthetic rehabilitation diabetic amputation”.

The reference lists of the retrieved articles were also reviewed (the ‘snowball method’) to identify additional studies of relevance to the topic. There were no limitations placed on the publication data to ensure comprehensive coverage. Only articles published in English were considered for inclusion.

### Inclusion Criteria

The studies selected for inclusion met the following criteria:

- The study population consisted of adult patients (≥18 years old) with diagnosed Diabetes Mellitus (Type 1 or Type 2).
- Patients underwent either minor LEA (toe, ray, transmetatarsal, partial foot amputation) or major LEA (below-knee, above-knee amputation).
- Studies reported at least one primary or secondary outcome related to LEA, including:
  - Mortality rates (perioperative, 30-day, 1-year, 5-year, or longer-term survival).
  - Reamputation incidence (risk or rates).
  - Postoperative complications (infection, wound healing disorders, ulcerations, etc.).
  - Energy expenditure or metabolic cost or ambulation post-amputation.
  - Functional outcomes, including rehabilitation status, prosthetic use, ambulation, mobility, and quality of life measures.
- Studies classified as observational (retrospective/prospective cohort studies), population-based studies, systematic reviews, or meta-analyses.
- Articles available in full-text format and published in English language journals.

### Exclusion Criteria

Studies meeting the following criteria were excluded from this review:

- Studies focusing exclusively on traumatic amputation (due to injury, trauma, military incidents).
- Studies exclusively involving non-diabetic populations.
- Case reports, conference abstracts, editorials, letters, or other non-original studies lacking primary clinical outcome data.
- Animal studies, basic laboratory research, or purely surgical technique-oriented publications without clinical outcomes.
- Studies without clear and relevant outcome measures related to mortality, reamputation, postoperative complications, or functional outcomes following LEA.

### Data Extraction and Synthesis

The relevant data were extracted systematically from each included study according to the following predefined categories:

- Study characteristics: authors, publication year, design, patient population, sample size, and study duration.
- Demographic data: age, sex distribution, comorbidities (e.g., renal disease, peripheral vascular disease, cardiovascular disease).
- Clinical intervention details: classification of amputation as minor or major, and the specific level of amputation.
- Primary outcomes: mortality and survival rates at various intervals post-amputation.
- Secondary outcomes:
  - Incidence and risk factors associated with reamputation.
  - Postoperative complications such as wound infections, stump ulcers, and skin issues related to prosthetic use.
  - Rehabilitation outcomes, including functional mobility, prosthetic fitting success, and energy expenditure during ambulation.

Given the narrative nature of this review, a formal methodological quality assessment tool was not employed. Nevertheless, articles were evaluated based on their methodological clarity, sample size, clinical relevance, and contribution to the overall understanding of the outcomes post-LEA in diabetic patients.

The findings from the included studies were synthesized narratively, with emphasis on identifying the common themes, important differences, clinical implications, and gaps within the existing literature. A total of 42 studies were included in this narrative review.

Quantitative values presented in the Results section represent the calculated averages, ranges, or representative estimates derived by the authors based on consistent findings across the included studies. This approach was adopted to provide a unified and interpretable synthesis of data from heterogeneous sources.

## Results

### Mortality Outcomes

Mortality rates following lower extremity amputation (LEA) in diabetic patients vary significantly based on the level and extent of amputation. Major amputations, particularly *Above-Knee Amputations* (AKA), consistently show substantially higher mortality rates than minor amputations. Short-term (30-day) mortality after major LEA ranges between 8% and 15%, while minor amputations demonstrate significantly lower short-term mortality, typically under 5% [3], [4]. These findings are consistent with large cohort studies showing high early postoperative mortality after major LEA in diabetic patients [4]. An overview of the mortality outcomes and hospitalization metrics across amputation levels is presented in Table 1.

**Table 1:** Mortality and hospitalization outcomes following lower extremity amputation (LEA)

Metric	Group A	Group B	Value/Comparison
Mortality Hazard Ratio	Diabetic patients	Non-diabetic patients	1.57 vs 1.00
Median Survival (Post-LEA)	Diabetic patients	Non-diabetic patients	27.2 months vs 46.7 months
Hospital Stay Duration	Major LEA	Minor LEA	31.8 ± 13.6 vs 16.2 ± 7.35 days
Time to Readmission (Median)	Diabetics with PVD	Non-diabetics	165 days vs 47 days

*Note*. The diabetic status significantly increases the hazard ratio and reduces the median survival. Hospital stay durations and readmission times are notably longer in major amputations and among diabetic patients with peripheral vascular disease. Surgical delays are associated with a measured increase in mortality risk.

Long-term survival differences are even more pronounced. The five-year survival rate following major LEA varies widely but generally remains low, ranging from approximately 10% to 48%, while patients undergoing minor amputations have notably higher five-year survival rates, typically between 29% and 69% [5], [6]. As summarized in Table 2, five-year survival rates range from 10 to 48% for major LEA and 29–69% for minor LEA. Similarly, Izumi et al. reported a poor long-term survival among diabetic patients following their first amputation, with a five-year mortality rate exceeding 50% [29]. Within major amputations, patients undergoing above-knee amputations experience greater mortality compared to those undergoing *Below-Knee Amputations* (BKA), with five-year survival rates notably lower for AKA patients [7].

**Table 2:** Survival rates at 30 days, 1 year, 3 years, 5 years, and 10 years following lower extremity amputation (LEA)

Amputation Type	30-Day	1-Year	3-Year	5-Year	10-Year
Major LEA (Overall)	11-15%	54-70.8%	30-41.3%	10-48%	0-10%
Minor LEA (Overall)	<5%	76-90.6%	60-72.8%	29-69%	20-58.5%
Above-Knee (AKA)	42-44%	~42%	-	16.7%	-
Below-Knee (BKA)	23-25%	~76%	-	33.7%	-
Ray/Toe Amputation	4.4% (10 mo)	-	-	15.8%	-

*Note*. Dashes (-) indicate time points not consistently reported across studies.

In addition to survival differences, hospitalization metrics also differ between the amputation types. Patients undergoing major LEA have a significantly longer average hospital stay, with durations averaging 31.8 ± 13.6 days, compared to 16.2 ± 7.35 days for patients undergoing minor LEA [8]. Furthermore, diabetic patients with *Peripheral Vascular Disease* (PVD) experience a markedly prolonged median time to hospital readmission post-discharge (165 days) compared to non-diabetic patients (47 days) [9].

Surgical timing is another critical factor influencing the mortality outcomes. Each additional day of delay before surgical amputation is associated with a 2% increase in mortality risk [10]. Thus, timely surgical intervention plays a pivotal role in improving the survival rates post-LEA.

Specific demographic trends have also emerged from the analysis. Younger diabetic men (under 45 years) experience unexpectedly higher mortality rates following major LEA compared to their female counterparts [11]. Conversely, among older patients (over 65 years), women exhibit higher mortality rates than men following similar procedures [11].

Additionally, the diabetic status itself substantially impacts the overall mortality outcomes. Diabetic patients exhibit a mortality *Hazard Ratio* (HR) of 1.57 compared to non-diabetic patients over a 12-year period [9]. The median survival following amputation in diabetic populations is notably shorter at approximately 27 months compared to about 47 months in non-diabetic patients [9].

In summary, the mortality outcomes strongly correlate with the amputation level, patient demographics, and diabetic status, thus highlighting the need for an early intervention and aggressive management strategies aimed at limb preservation wherever possible. Notably, the survival rates following LEA in diabetic patients have been reported to be comparable to those seen in malignant diseases [32].

### Reamputation Rates

Reamputation remains a significant concern in diabetic patients undergoing lower extremity amputations. The cumulative incidence of reamputation after the first amputation is substantial. One study reported a reamputation rate of 26.7% within the first year, which increases to 48.3% at three years and to 60.7% at five years [29], [12].

Several factors influence the risk of reamputation. *Peripheral Arterial Disease* (PAD) is among the most significant predictors, particularly contributing to contralateral limb loss in patients who initially undergo a major LEA. Inadequate vascular supply, poor wound healing, and infections are all common clinical pathways that lead to reamputation [30], [12], [13].

Patients undergoing minor amputations generally have a lower risk of reamputation compared to those undergoing major amputations, especially when aggressive preventative care and monitoring is employed [30]. Effective multidisciplinary care, prompt revascularization, glycemic control, and close wound surveillance are key strategies to reduce the reamputation rates and improve the overall limb salvage outcomes [14]. The cumulative reamputation trends are summarized in Table 3.

**Table 3:** Reamputation rates at 1, 3, and 5 years following lower extremity amputation (LEA)

Time Post-Amputation	Reamputation Rate
1 year	26.7%
3 years	48.3%
5 years	60.7%

### Risk Factors for Mortality and Complications

Multiple patient- and procedure-related factors contribute to an increased mortality and postoperative complications following lower extremity amputations in diabetic individuals.

Peripheral arterial disease (PAD) and *Chronic Kidney Disease* (CKD) are among the strongest predictors of poor outcomes, including both mortality and the need for reamputation. PAD is frequently associated with delayed wound healing, increased infection rates, and progression to higher-level amputations [33], [34], [35]. Similarly, vascular disease, infections, and delayed wound healing have been independently associated with worsened postoperative outcomes following LEA [33]. CKD significantly worsens the survival, especially in patients with end-stage renal disease or those on dialysis [34], [35].

The age and sex also influence the outcomes. Older patients, especially those over 70 years, exhibit higher perioperative mortality and complication rates [11], [34]. Women, particularly in the elderly group, experience worse outcomes compared to men following both minor and major LEA [11].

Glycemic control and the presence of diabetic foot infections play a critical role in complications. Poorly controlled diabetes contributes to microvascular compromise and a reduced immune response, increasing the likelihood of surgical site infections and impaired wound healing [33].

Delay in surgery has also been identified as a modifiable risk factor. Each additional day between the admission and the amputation surgery is associated with a 2% increase in the risk of mortality [18]. Extended preoperative hospital stays are linked to an elevated infection risk and a worsened rehabilitation potential post-amputation [34], [35]. Moreover, postoperative strategies such as continuous peripheral nerve blocks have been associated with an enhanced recovery, reduced pain, and potentially shorter hospital stays following the amputation [39]. Together, these risk factors emphasize the importance of a proactive, multidisciplinary care approach, prioritizing an early diagnosis, revascularization, and infection control to improve the patient outcomes and reduce the amputation-associated mortality.

### Functional Outcomes and Rehabilitation

Functional recovery after lower extremity amputation in diabetic patients is influenced by the level of amputation, the presence of comorbidities, and the rehabilitation access. Patients with minor amputations tend to retain greater mobility and independence compared to those undergoing major amputations [20].

Energy expenditure during ambulation is markedly increased in individuals with major amputations. Below-knee amputees require approximately 10–40% more energy to walk than non-amputees, while above-knee amputees may expend up to 65% more energy [21], [22]. This significantly impacts the endurance and quality of life, especially in the elderly or those with multiple comorbidities.

Prosthetic use is more successful in patients with lower-level amputations. Individuals with transmetatarsal or below-knee amputations have higher rates of prosthetic acceptance and functionality. In contrast, above-knee amputees often face challenges with prosthetic use due to energy demands, stump complications, or impaired balance [23]. Furthermore, prosthetic use itself can lead to complications such as residual limb skin breakdown and ulceration, which significantly impact long-term prosthetic function and patient mobility [42]. Rehabilitation outcomes also depend on early prosthetic fitting and access to multidisciplinary rehabilitation programs. The timely initiation of the physical therapy, psychological support, and occupational therapy improves the mobility outcomes and the overall reintegration into daily activities [24], [25].

Additionally, the mean postoperative survival differs by the amputation level, with transtibial (BKA) amputees demonstrating a longer average survival (13.6 months) compared to transfemoral (AKA) amputees (7.1 months) [26].

Overall, efforts to preserve the limb length, ensure an early rehabilitation, and manage comorbidities are essential to maximizing functional outcomes post-amputation in diabetic patients. A summary of functional outcomes and energy expenditure at different LEA levels is provided in Table 5.

**Table 4:** Factors associated with an increased risk of mortality following LEA. Effect estimates are reported as *Hazard Ratios* (HR), *Odds Ratios* (OR), or *Mortality Rate Ratios* (MRR), depending on the source study

Risk Factor	Comparison / Stratification	Quantified effect / Observation
Calcaneal Lesion	Present vs Absent	OR = 4.52 for major LEA
Wagner Grade 5 Ulcer	Grade 5 vs Lower grades	OR = 4.57 for major LEA
Arterial Insufficiency	Present vs Absent	OR = 4.43 for major LEA
Age < 45	Younger men vs Younger women	46.5 vs 0 per 1000 PY post-major LEA
Age > 65	Older women vs Older men	246.6 vs 195.7 per 1000 PY post-major LEA
Chronic Kidney Disease (CKD)	CKD vs No CKD	Associated with significantly increased mortality and complications
Peripheral Arterial Disease	PAD vs No PAD	Associated with higher reamputation and mortality
Surgical Delay	Per 1-day delay	+2% increased mortality per additional day (HR = 1.02)
Poor Glycemic Control	Uncontrolled vs Controlled diabetes	Increased infections, poor healing, increased reamputation risk
Diabetic Foot Infection	Infected vs Non-Infected patients	Higher complication rates and worse outcomes

**Table 5:** Functional and survival outcomes following lower extremity amputation (LEA) in diabetic patients

Outcome	Finding
Energy Expenditure (BKA vs Non-Amputees)	+10-40% increased during ambulation
Energy Expenditure (AKA vs Non-Amputees)	+65% increased during ambulation
Prosthetic Success	Higher with minor LEA and BKA; lower with AKA
Functional Mobility	Better in transtibial (BKA) vs transfemoral (AKA)
Mean Survival Post-Transfemoral Amputation	7.1 months
Mean Survival Post-Transtibial Amputation	13.6 months

*Note*. Energy expenditure values represent estimated increases during ambulation based on standard rehabilitation studies.

## Discussion

This narrative review highlights the substantial differences in clinical and functional outcomes between minor and major lower extremity amputations (LEA) in diabetic patients. Major LEAs – particularly above-knee amputations (AKA) – are consistently associated with significantly higher mortality rates, prolonged hospital stays, an increased risk of reamputation, and poorer functional recovery compared to minor amputations [28], [32], [34]. In contrast, minor LEAs are more likely to preserve the limb function, facilitate prosthetic use, and are linked to higher survival rates and an improved quality of life [31].

These findings align with the existing literature, demonstrating that minor amputations yield better postoperative outcomes and improve lower energy demands during ambulation [9], [10]. Conversely, patients undergoing major amputations often experience profound physical limitations and are more susceptible to psychological distress, including depression, fear of further amputation, and reduced life satisfaction [27], [35], [41]. These outcomes emphasize the need for early limb-salvage strategies and comprehensive, patient-centred care that includes psychosocial support.

Multiple clinical factors contribute to poorer outcomes in patients undergoing major LEAs. Poor glycemic control, peripheral arterial disease (PAD), chronic kidney disease (CKD), and active infection have been independently associated with an increased mortality and complication rates [33], [37]. These risk factors highlight the importance of stringent metabolic control, early detection of vascular insufficiency, and timely revascularization to prevent the progression to high-level amputation [36].

Reamputation remains a major concern in diabetic foot management, with cumulative rates exceeding 60% within five years of the initial procedure [12]. This underscores the chronic and recurrent nature of diabetic foot disease. Implementing multidisciplinary care – engaging diabetologists, vascular surgeons, podiatrists, and rehabilitation teams – is essential to improving the limb salvage, reducing mortality, and enhancing long-term outcomes.

This review has several limitations. As a narrative review, it is inherently subject to selection bias and lacks formal quality assessment of the included studies. Furthermore, heterogeneity in the study designs, inconsistent definitions of ‘minor’ and ‘major’ amputations, and variability in the outcome measures limit the direct comparability of the findings.

Future research should aim to develop validated risk stratification tools to identify patients at the highest risk for major amputation or reamputation [33]. Promising surgical advancements, such as *Targeted Muscle Reinnervation* (TMR), may reduce post-amputation pain and enhance the prosthetic function [38]. Additionally, innovation in the prosthetic technology, individualized rehabilitation strategies, and structured psychosocial support systems will be pivotal in optimizing functional recovery and the quality of life in this high-risk population.

## Conclusion

Early intervention and integrated, multidisciplinary care are critical to improving outcomes in diabetic patients facing lower extremity amputation. Emphasis on the limb preservation, aggressive management of comorbid conditions, and an expanded access to rehabilitation and psychological support can substantially reduce the burden of diabetes-related amputations. By adopting a proactive, patient-centred approach, healthcare systems can improve both the survival and the functional recovery in this vulnerable population.
